# Cytogenetics of two hylid frogs from Brazilian Cerrado

**DOI:** 10.1590/1678-4685-GMB-2017-0382

**Published:** 2018-11-29

**Authors:** Cíntia Pelegrineti Targueta, Vinícius Guerra, Priscilla Guedes Gambale, Rogério Pereira Bastos, Daniela de Melo e Silva, Mariana Pires de Campos Telles

**Affiliations:** ^1^ Laboratório de Genética & Biodiversidade, Departamento de Genética, Instituto de Ciências Biológicas, Universidade Federal de Goiás, Goiânia, GO, Brazil; ^2^ Programa de Pós-Graduação em Ecologia de Ambientes Aquáticos Continentais, Universidade Estadual de Maringá, Maringá, PR, Brazil; ^3^ Departamento de Ecologia, Instituto de Ciências Biológicas, Universidade Federal de Goiás, Goiânia, GO, Brazil; ^4^ Laboratório de Mutagênese, Departamento de Genética, Instituto de Ciências Biológicas, Universidade Federal de Goiás, Goiânia, GO, Brazil; ^5^ Escola de Ciências Agrárias e Biológicas, Pontifícia Universidade Católica de Goiás, Goiânia, GO, Brazil

**Keywords:** Anura, karyotype, Ololygon, Scinax

## Abstract

Cytogenetic data can be useful for taxonomic and phylogenetic studies, as well as to provide information about chromosome evolution. Therefore, it may help design conservation priorities for some threatened species, such as anurans. Herein, we describe the karyotypes of *Scinax constrictus* and *Ololygon centralis*, native endemic species from the Brazilian Cerrado. Chromosome preparations for both species were stained with Giemsa for morphological analyses and then impregnated by the Ag-NOR method for localization of the nucleolar organizer region (NOR). Both species had 24 chromosomes, as confirmed by meiotic analyses, which showed 12 bivalents. Chromosome morphologies presented the same pattern for *Scinax* and *Ololygon* compared to species already karyotyped in both genera. The NOR was interstitially located in the long arm of pair 7 in *S. constrictus*, whereas in *O. centralis* it was found near the centromere in the long arm of pair 1, thus diverging from what is commonly found for other *Ololygon* species. Therefore, we provide the first description of the karyotype of *O. centralis* and the first report of the localization of the NOR for the karyotype of both species. Our study increases the cytogenetic knowledge in species of the genera *Scinax* and *Ololygon*, and provide support for further studies on the taxonomy, ecology, and evolution of hylid anurans.

The tree frog family Hylidae is the richest in number of species among anurans, with 716 described species (Frost, 2018). It comprises seven subfamilies: Acridinae (21 spp.), Comphomantinae (185 spp.), Dendropsophinae (107 spp.), Hylinae (167 spp.), Lophyohylinae (85 spp.), Pseudinae (13 spp.), and Scinaxinae (137 spp.). Scinaxinae comprises now four genera: *Julianus* (2 spp.), *Ololygon* (48 spp.), *Scinax* (72 spp.), and *Sphaenorhynchus* (15 spp.) (Duellman *et al.*, 2016). The *Ololygon* species were previously considered to belong to the genus *Scinax*, in the *Scinax catharinae* clade, while species that remained in the genus *Scinax* comprise the *Scinax ruber* clade (Faivovich, 2002; Faivovich *et al.*, 2005). The species of Scinaxinae are distributed throughout Central and South America, from southern and eastern Mexico to Uruguay and northern Argentina (Frost, 2018).

Knowledge on chromosome number and morphology of hylids is still sparse, and in Scinaxinae, only 45 species of the genera *Scinax* and *Ololygon* have known karyotypes (Peixoto *et al.*, 2015, 2016). The karyotypes of *Scinax* and *Ololygon* analyzed so far are very conservative concerning the diploid number (2n=24), although morphological patterns can be discerned among them (Bogart, 1973; Nunes and Fagundes, 2008; Oliveira *et al.*, 2010; Cardozo *et al.*, 2011; Nogueira *et al.*, 2015; Peixoto *et al.*, 2015). The localization of the nucleolar organization region (NOR) and the heterochromatic pattern were described for most of the species analyzed. For most of them, the NOR was observed on chromosome pair 11 for *Scinax* species and on pair 6 for *Ololygon* species (species previously grouped in the *Scinax catharinae* clade by Faivovich *et al.*, 2005) (Cardozo *et al.*, 2011), and C-banding patterns were predominantly centromeric (Cardozo *et al.*, 2011; Peixoto *et al.*, 2015).

The *Scinax rostratus* group was recognized by Faivovich *et al.* (2005), in which *Scinax constrictus* is allocated (Lima *et al.*, 2004). Pombal-Jr and Bastos (1996) described *Ololygon* (=*Scinax*) *centralis* and allocated it inside the *Scinax catharinae* group according to its morphological and acoustic data. Both species are endemic to the Brazilian Cerrado, which is considered a biodiversity hotspot, with high levels of endemic species, most of them being threatened by anthropic activities (Myers *et al.*, 2000; Mittermeier *et al.*, 2005). In addition, the Cerrado has been reported to house 52% of endemic anuran species (Valdujo *et al.*, 2012). Although several studies have demonstrated the recent degradation and loss of biodiversity (Beuchle *et al.*, 2015; Françoso *et al.*, 2015), many species have been described for this biome (e.g., Berneck *et al.*, 2017; Haga *et al.*, 2017).

Little is known about the cytogenetics of frogs in the Cerrado. An increase in knowledge, therefore, may help to improve studies on the taxonomy, phylogeny, and evolution of anurans in this threatened biome. Herein, we describe the chromosome number and NOR pattern in several populations of *Scinax constrictus* and *Ololygon centralis*, two endemic hylids from the Brazilian Cerrado.

The field work was conducted at three different localities of the Cerrado (Figure 1; Table S1). We used the sampling method involving visual and acoustic searches (Heyer *et al.*, 1994) to find the individuals. After being found, we captured the individuals with our hands and carefully placed them in plastic bags, and later they were maintained in a Styrofoam box for transport to the laboratory. Individual specimens received an application of colchicine and after four hours, specimens were euthanized with 5% lidocaine. The liver and tissues from the hind paw muscles, intestine, and glands were removed for molecular analysis. Subsequently, each specimen was fixed with 10% formalin, preserved in 70% alcohol, and deposited in the Coleção Zoológica of the Universidade Federal de Goiás (ZUFG), Brazil (Table S1). Sampling dates, capture and species collection were authorized under a license granted by the Instituto Chico Mendes de Conservação da Biodiversidade, Brazil, (ICMBio; number 46522-3), and the experiments were authorized by the Comitê de Ética ao Uso de Animais of the Universidade Federal de Goiás (number 109/14).

**Figure 1:**
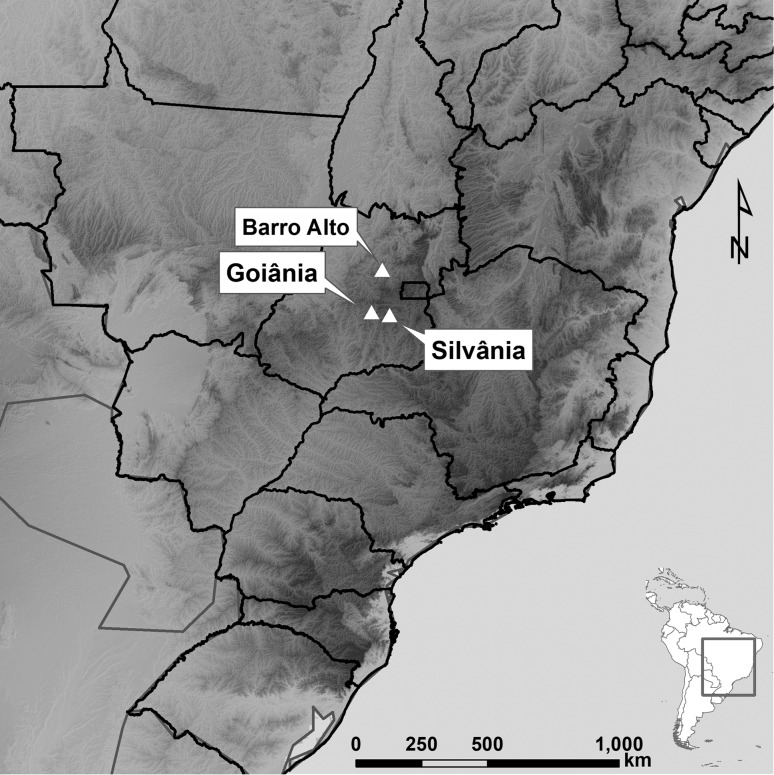
Geografic view of *Scinax constrictus* and *Ololygon centralis* sampled sites. *Scinax constrictus* were collected at Barro Alto and Goiânia, GO. *Ololygon centralis* were found at its type-locality, Floresta Nacional de Silvânia, Silvânia, GO.

We captured samples of *Scinax constrictus* and *Ololygon centralis*. *S. constrictus* (Lima *et al.*, 2005) is a small-sized hylid (26.09 ± 1.83 mm for males, 31.93 ± 2.53 mm for females; Lima *et al.*, 2005) distributed in the Brazilian states of Goiás, Tocantins, Minas Gerais, and Mato Grosso do Sul (Frost, 2017). The reproduction of this species occurs during the rainy season (September–February) in the Cerrado Biome (Gambale *et al.*, 2014). Males may be found in lentic water bodies and the social behavior is mediated mainly by acoustic signals (Gambale *et al.*, 2014). Information on some aspects on the ecology of this species can be found in the studies of Lima *et al.* (2005) and Gambale *et al.* (2014). Males of *S. constrictus* were collected at two distinct sites: four males were from Barro Alto, Goiás (ZUFG9310, ZUFG9311, ZUFG9312, ZUFG9319) and 10 males from Goiânia, Goiás (ZUFG8715, ZUFG8716, ZUFG8717, ZUFG8718, ZUFG8719, ZUFG8720, ZUFG8721, ZUFG8722, ZUFG8723, ZUFG8724).

*Ololygon centralis* (Pombal and Bastos, 1996) is a small-sized hylid frog (19.74 ± 0.87 mm for males; Pombal-Jr and Bastos, 1996) distributed in the Distrito Federal and Goiás State, Brazil (Frost, 2017). The reproduction of this species occurs mainly during the rainy season (January–April) in the Cerrado Biome (Bastos *et al.*, 2011). Males may be found in lotic water bodies, and the social behavior is mediated by acoustic signals (Bastos *et al.*, 2011). Eleven specimens of *O. centralis* were collected, of which four were females (ZUFG9294, ZUFG9301, ZUFG9303, ZUFG9304) and seven were males (ZUFG9295, ZUFG9296, ZUFG9297, ZUFG9298, ZUFG9299, ZUFG9300, ZUFG9302) from the type-locality Floresta Nacional de Silvânia (FLONA), Silvânia, Goiás, Brazil.

Chromosome preparations were obtained from intestinal and testis cells of the animals previously treated with colchicine for 4 hours following the method of Schmid (Schmid, 1978; Schmid *et al.*, 1979) with minor modifications. The cells were maintained at -20 °C. For morphological analyses, the cell suspensions were dropped onto clean slides and submitted to conventional staining with 10% Giemsa. The localization of the nucleolar region was detected by the Ag-NOR method described by Howell and Black (1980). Mitotic and meiotic chromosomes were photographed under a Leica microscope and with the LAS EZ software (Leica). The chromosomes were ordered with Photoshop CS5 and classified following Green and Sessions (1991).

After analyses, all males of *S. constrictus* showed a diploid number with 24 chromosomes. Mitotic chromosome pairs 1, 2, 9, 10, 11, and 12 were characterized as metacentric, while the chromosome pairs 3, 4, 5, 6, 7, and 8 were characterized as submetacentric (Figure 2, Table 1). The NOR was detected at the interstitial region of the long arm of pair 7, in the same place where a secondary constriction is presented (Figure 2). The diploid number was confirmed by the presence of 12 bivalents in the meiotic preparations (Figure 2). In addition, all specimens of *O. centralis*, i.e., both the males and females, showed 2n=24 chromosomes, in which the pairs 1, 2, 3, 4, 5, 6, and 7 were submetacentric, while pairs 8, 9, 10, 11, and 12 were metacentric (Figure 3, Table 1). The NOR was detected at the proximal region of the long arm of pair 1 (Figure 3). The diploid number was confirmed by the presence of 12 bivalents in the meiotic preparations (Figure 3).

**Figure 2:**
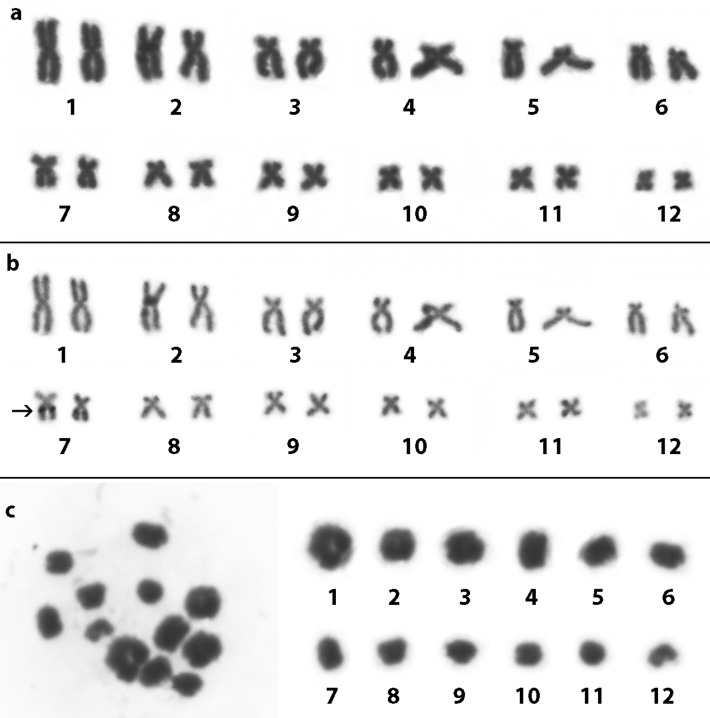
Karyotype of *Scinax constrictus* (ZUFG8715) stained with 10% Giemsa (a), Ag-NOR impregnation (ZUFG9310) (b) and meiotic bivalents (ZUFG8718) (c) The arrow in (b) shows the localization of the NOR.

**Figure 3:**
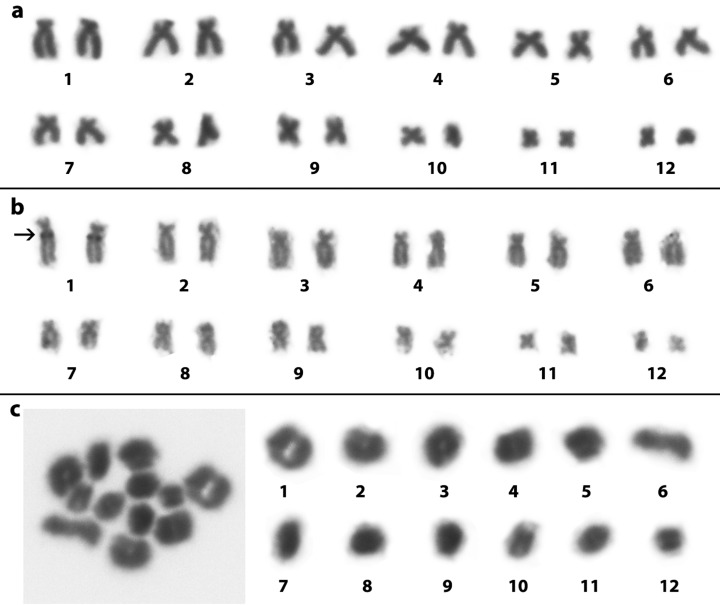
Karyotype of *Ololygon centralis* (ZUFG9300) stained with 10% Giemsa (a), Ag-NOR impregnation (ZUFG9304) (b) and meiotic bivalents (ZUFG9295) (c) The arrow in (b) shows the localization of the NOR.

**Table 1:** Morphometric parameters of *Scinax constrictus* and *Ololygon centralis* karyotypes. Chromosome classification followed Green and Session (1991). (m) metacentric; (sm) submetacentric; (st) subtelocentric; (t) telocentric.

*Scinax constrictus*												
Chromosome number	1	2	3	4	5	6	7	8	9	10	11	12
Relative Length (%)	13.20	11.65	10.85	9.89	9.04	8.27	7.85	7.06	6.31	5.96	5.40	4.52
Arm ratio	1.32	1.56	1.75	2.30	2.34	2.14	1.98	1.86	1.40	1.38	1.29	1.24
Classification	m	m	sm	sm	sm	sm	sm	sm	m	m	m	m
*Ololygon centralis*												
Chromosome number	1	2	3	4	5	6	7	8	9	10	11	12
Relative Length (%)	11.58	10.64	9.92	9.56	9.22	8.98	8.47	7.93	7.21	6.08	5.56	4.85
Arm ratio	2.94	2.65	2.48	2.38	2.22	2.13	2.17	1.60	1.31	1.33	1.28	1.31
Classification	sm	sm	sm	sm	sm	sm	sm	m	m	m	m	m

The diploid number (2n=24) described for *S. constrictus* and for *O. centralis* is the same number previously described for species of both genera (Bogart, 1973; Nunes and Fagundes, 2008; Oliveira *et al.*, 2010; Cardozo *et al.*, 2011; Nogueira *et al.*, 2015; Peixoto *et al.*, 2015). Within the Scinaxinae family, as proposed by Duellman *et al.* (2016), the diploid number seems to be conserved (2n=24), changing only for *Sphaenorhyncus caramaschii* (Suárez *et al.*, 2013), which shows a karyotype composed of 26 chromosomes. However, the diploid number also differs when compared to closely related species to Scinaxinae (Duellman *et al.*, 2016), such as those of the genera *Phyllodytes* and *Scarthyla* with 2n=22, *Aparasphenodon*, *Trachycephalus*, *Itapotihyla*, *Osteocephalus*, *Pseudis* (except one species with 2n=28), and *Lysapsus* with 2n=24, and the genus *Dendropsophus* with 2n=30 (Busin *et al.*, 2001, 2006; Kasahara *et al.*, 2003; Gruber *et al.*, 2012; Suárez *et al.*, 2013).

Notably, Oliveira *et al.* (2010) found only 22 chromosomes for *Scinax constrictus*, which represents the first report of a different diploid number within the genus, suggesting a case of population variation. However, the chromosomal morphology was very similar to that found in the present study. Hence, this improved knowledge on the population cytogenetics will contribute with the elucidation of the possible evolutionary differentiation among populations of *S. constrictus*. Moreover, the chromosome morphology assigned for *Scinax* (= *Scinax rube*r clade) and *Ololygon* (= *Scinax catharinae* clade) by Bogart (1973) was also now detected for *S. constrictus* and *O. centralis*, respectively, as well as in some other previous studies (Oliveira *et al.*, 2010; Cardozo *et al.*, 2011; Nogueira *et al.*, 2015; Peixoto *et al.*, 2015, 2016; Lourenço *et al*., Gruber *et al.*, 2017). All these findings demonstrate that chromosomal morphology is a conserved feature in both of these Scinaxinae genera.

The nucleolar organizer region (Ag-NOR) was localized herein for the first time for the karyotypes of *S. constrictus* and *O. centralis*. It was detected in the long arm of pair 7 in *S. constrictus*, thus differing from previous data for *Scinax* and *Julianus* species (previously, *Scinax ruber* clade), where they are found in the short arm of pair 11 (Cardozo *et al.*, 2011; Nogueira *et al.*, 2015; Peixoto *et al.*, 2016; Gruber *et al.*, 2017). Notably, the NOR was also found in this same chromosome pair in phylogenetically more distant species, such as those of the genera *Xenohyla*, *Lysapsus*, and *Pseudis* (except for one population). In those cases, the chromosome was morphologically similar to the pair 7 of *Scinax constrictus* (Busin *et al.*, 2001, 2006; Suárez *et al.*, 2013). In addition, the position of the NOR found in *S. constrictus* resembles more that found in species from other genera than in species of *Scinax* itself, which could represent a plesiomorphic state maintained by *S. constrictus,* or even a resurrected feature during the evolution of the group. In this sense, the increased characterization of the *Scinax rostratus* group karyotype, in which *S. constrictus* is allocated, should help to further understand the NOR evolution within the Scinaxinae family.

Most of the *Ololygon* (= *Scinax catharinae* clade) species already karyotyped have the NOR localized at the pericentromeric region of the short arm of pair 6 (Cardozo *et al.*, 2011; Peixoto *et al.*, 2015; Gruber *et al.*, 2017). However, the chromosome bearing the NOR in *O. centralis* is pair 1. This is the only species within the Scinaxinae subfamily that has the NOR located in this chromosome pair, corresponding to a probable autapomorphy for this species. Moreover, for *O. caissara*, chromosome 2 is the NOR bearing one (Lourenço *et al.*, 2016). Due to the very similar morphology shared by pairs 1 and 2 of *Ololygon* species, additional studies are needed to conclude whether these two species share the same NOR position, or if this is an acquired, particular evolutionary characteristic.

Despite analyzing males and females, we did not detect any sex chromosomal differentiation or NOR heteromorphism in *O. centralis*. Cardozo *et al.* (2011) identified one heteromorphic pair related to the presence of the NOR in *Ololygon canastrensis*. In this case, males and females presented the NOR in the terminal region of the long arm of pair 11, but females had an additional NOR in the short arm of one homologue of pair 6 (Cardozo *et al.*, 2011). In addition, Peixoto *et al.* (2016) also detected another case of NOR variation in *Ololygon tripui*, but this was not sex related. In that case, besides the Ag-NOR location in pair 6 of the karyotype, rDNA sites were also evidenced in both the third and the fourth pairs by fluorescence *in situ* hybridization (FISH). Although a more conserved NOR state in some Anura groups is observed, variations on its position and number are widely described, even between individuals of the same species (Schmid *et al.*, 1995; Silva *et al.*, 1999; Targueta *et al.*, 2010; Bruschi *et al.*, 2013). The presence of multiple NOR sites, even in the same species or populations of organisms, may be explained by several cellular events, such as duplication of repeats, transposition, ectopic recombination, or chromosome rearrangements (Schmid, 1982; Hall and Parker, 1995; LeRiche *et al.*, 2015).

In conclusion, this study reports additional karyotype characteristics of two endemic anuran species from the Brazilian Cerrado Biome. It emphasizes that the chromosome morphology is conserved within both genera, but that they show a specific karyotype organization and NOR location, providing support for the taxonomic changes proposed by Duellman *et al.* (2016) based on molecular data. However, as the Scinaxinae subfamily is a species-rich group, further studies on the most related species are needed to better understand the evolutionary cytogenetics of this Hylidae taxon.

## Supplementary material

The following online material is available for this article:

Table S1 - Data from collected specimens.
