# Supplementary material for: Cytogenetics of two hylid frogs from Brazilian Cerrado
Source: Genet Mol Biol. 2018 Nov 29;41(4):814–9. doi: 10.1590/1678-4685-GMB-2017-0382 (PMC6415605; doi:10.1590/1678-4685-GMB-2017-0382)
Supplement: Supplementary file 1 [file 1415-4757-GMB-1678-4685-GMB-2017-0382-s001.pdf]

Supplementary to "Cytogenetics of two hylid frogs from Brazilian Cerrado"

Table S1 - Data from collected specimens, containing the locality, number of specimens, gender, and voucher from Coleção Zoológica of Universidade Federal de Goiás (ZUFG).

Species	Sampled site	N	Gender	Vouchers
Scinax constrictus	Barro Alto ─ GO (15° 3'49.00"S, 48°53'34.00"W, SAD69)	4	Male	ZUFG9310, ZUFG9311, ZUFG9312, ZUFG9319
	Goiânia ─ GO (16°31'55.4"S, 49°16'35.3"W, SAD69)	10	Male	ZUFG8715, ZUFG8716, ZUFG8717, ZUFG8718, ZUFG8719, ZUFG8720, ZUFG8721, ZUFG8722, ZUFG8723, ZUFG8724
Ololygon centralis	Silvânia ─ GO (16°38'04.4"S, 48°39'31.2"W, SAD69)	4	Female	ZUFG9294, ZUFG9301, ZUFG9303, ZUFG9304
		7	Male	ZUFG9295, ZUFG9296, ZUFG9297, ZUFG9298, ZUFG9299, ZUFG9300, ZUFG9302
